# EHMTI-0337. Visual evoked potential abnormalities in migraineurs: is there a correlation with alpha power and photic driving on the EEG?

**DOI:** 10.1186/1129-2377-15-S1-E16

**Published:** 2014-09-18

**Authors:** D Magis, R Baschi, S Sava, E Vecchio, J Schoenen

**Affiliations:** 1 University Department of Neurology, Headache Research Unit, Liege, Belgium

## Background

Interictally, episodic migraine is associated with reduced habituation of visual evoked potentials (PR-VEP) due to decreased thalamic drive and cortical preactivation. EEG studies performed in migraineurs interictally demonstrated increased "photic driving" at high frequency visual stimulation, attributed to cortical hyperexcitability.

## Aim

To compare for the first time in the same migraine patients EEG power spectra at rest and during photic stimulation with PR-VEP characteristics.

## Method

Thirty-seven episodic migraineurs underwent EEG and PR-VEP recordings in random order. The power spectra of occipital EEG activity were measured during a 2-min recording at baseline and during two 15-sec trains of 20Hz flash stimulation.

## Results

Twenty-two patients were in interictal and 15 in(peri)ictal phase. As described before, PR-VEP habituation tended to be reduced between attacks and overall the habituation deficit was associated with lower initial PR-VEP amplitude.

Between attacks, power of the 20Hz frequency band during photic stimulation, i.e. photic driving, tended to be lower (p=0.08) and baseline alpha power higher (p=0.06). Photic driving was also positively correlated with PR-VEP amplitudes and negatively with PR-VEP habituation (p<0.05).

During attacks, alpha power was associated with increased PR-VEP habituation (p<0.05).

## Conclusion

We show that the interictal PR-VEP habituation deficit is associated with low photic driving at 20 Hz. During the attack, both habituation and photic driving increase compared to interictal state. Both tests are thus consistent and confirm that visual cortex preactivation decreases between attacks. Moreover, our correlations corroborate that during migraine attack visual cortex habituation increases proportionally to cortical preactivation and enhanced thalamic drive.

No conflict of interest.

